# Z-back-cut cheiloplasty: a cadaveric and clinical study on lip lengthening in unilateral cleft lip repair as a proof of concept

**DOI:** 10.1186/s40902-025-00470-z

**Published:** 2025-07-07

**Authors:** Till Wagner, Marloes Nienhuijs, Stefaan Berge, Dietmar Ulrich

**Affiliations:** Radboud University Nijmegen Medical Centre, Nijmegen, Netherlands

**Keywords:** Unilateral cleft lip, Incision line, Cleft lip closure, Incision pattern of unilateral cleft lips, Skin tension, Z-plasty

## Abstract

**Background:**

The clinical outcome of unilateral cleft lip closure depends on both the incision pattern and vertical scar retraction as well as complete lip muscle closure. Existing techniques focus on the lengthening of the cleft side with reducing visible scarring in addition to correct muscle adaptation but are still struggling with scar contraction. We aimed to optimize clinical results by developing a new incision design integrating benefits of established techniques with our own considerations.

**Methods:**

A study—using 10 adult cadavers—compared two vertical incision lines: the Millard advancement-rotation flap and our Z-back-cut cheiloplasty which reassembles a Z-plasty at the nostril by combining with a back cut at the lower columella rim. A skin distraction model assessed the impact of tension on lengthening. Our technique demonstrated increased vertical height compared to Millard’s method. Based on these findings, we applied our approach in clinical settings.

**Results:**

The Millard flap showed significantly less elongation (up to 35%) between the lateral cupid’s bow and the columella base than our method. This suggests that the Z-plasty principle is beneficial in unilateral cleft lip closure. Clinical outcomes confirmed its applicability in both partial and complete clefts.

**Discussion:**

Applying our findings in pediatric patients yielded at least equivalent results to the Millard II technique, even in cases with postoperative wound infections and increased contraction risk.

**Conclusion:**

Our research supports integrating Z-plasty principles into unilateral cleft lip repair. We plan to use this technique in future surgeries where indicated.

## Introduction

Long-term results in unilateral cleft lip surgery depend on the visibility of the remaining scar and the scar retraction, which often creates an unpleasant whistling deformity. However, the most important procedure for a pleasing result in cleft lip surgery is a complete mobilization of the orbicularis muscle and its exact adaptation before starting the skin suture. Retraction is the most common issue addressed by different incision patterns for lengthening of native skin at the cleft side of the philtrum. The most commonly used technique of Millard [1], and its derivatives, such as of Mohler [2], create a less appealing scar below the lower nostril due to the c-flap. Millard’s technique, as well as other approaches like Murawski’s [3], where part of the incision line runs below or across the basal central part of the columella, also lead to more visible scarring. Alternative techniques, such as Pfeifer’s, create a straight line with minimal scarring but are more often associated with scar contraction and whistling deformities [4]. The Tennison-Randall technique has a major drawback: disruption of the philtrum by disregarding the aesthetic subunit borders of the upper lip [5]. Similarly, Fisher’s exact subunit technique [6] has disadvantages, including lateral lip tissue scarification [7]. Even in 2024, the search for optimized incision patterns in cleft lip surgery remains ongoing [8]. Dissatisfied with our long-term results using the Millard 2 technique, we sought to develop a technique that combines effective lip lengthening with reduced visible scarring and minimized lip retraction. To interrupt the vertical retraction vector and lengthen the lip, we incorporated the Z-plastic principle at a less conspicuous location. By screening the literature from the past 20 years, we also integrated Fisher’s skin triangle [6, 9], and the transverse mucosal release technique described by Chiang et al. [10] and Baek et al. [11].

## Material and methods

### Material

**Fig. 1:**
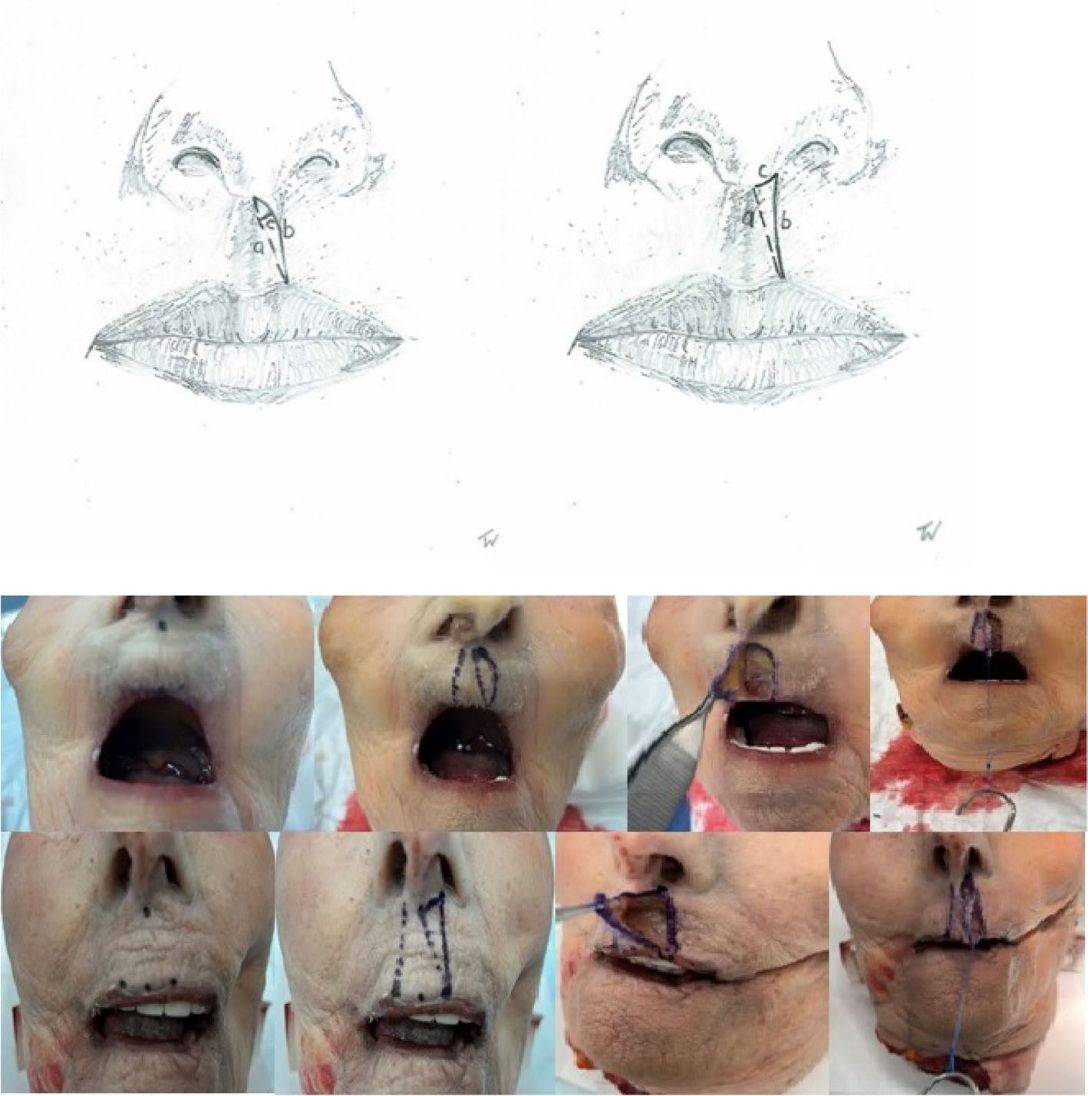
Incision lines in our cadaver model; left according to Millard (line **b**) and right with a straight line with a back-cut (line **c** and **b**). Drawing of Millard incision, back-cut incision, and tension force lengthening test. Starting point of line a on the left: Center of the base of the columella. End point of line **a** on the left: highest lateral point of the cupid’s bow. Line **b** on the left: identical start and end points. Line **c** on the left represents the maximum distance between line **a** and line **b**. Line **a** on the right is identical to the left side, starting point of line **c** is as for line **a**, but runs along the lower base of the columella to its lateral edge and extends caudally to the end point of line a on the right

After screening the relevant literature, we conducted a cadaver study to gain theoretical insights into the physical properties of skin distraction. No previous study has demonstrated, in a cadaveric model, the extent of lip lengthening achievable with Millard’s technique compared to others. Ten intact adult cadaveric heads, without any signs of trauma, were obtained from the willed donor program at the affiliated medical university cadaver lab. Age and nationality were unknown. Pediatric heads were unavailable; therefore, the effect of aging skin on elasticity could not be measured. When searching actual public databases for studies on biomechanical properties of the skin, we found a recent comprehensive review paper on this topic with the main result that aging skin becomes thinner, stiffer, less tense, and less flexible. Skin tension measured during in vivo uniaxial load and elasticity modules are higher in children than in elderly adults. Mean ultimate skin deformation before bursting is 75% for newborns and 60% for the elderly [12]. From this, we conclude that there is a certain comparability between the skin of old and young people. All specimens were fresh-frozen and defrosted immediately before dissection. In five of the ten specimens, standard Millard advancement-rotation flaps were dissected under loupe magnification on the left side of the philtrum. In the remaining five specimens, back-cut incision flaps were raised as part of our designated Z-back-cut cheiloplasty. We measured the length of the incision lines in Millard’s advancement-rotation flap (see Fig. 1a–c, left) and our back-cut incision (see Fig. 1a–c, right) under varying tension forces (see Fig. 1, below). Line a runs from the center of the lower base of the columella in a straight line to the highest lateral point of the Cupid’s bow. Line b runs from the same starting point to the identical end point, but according to Millard’s incision technique, and line c represents the maximum distance between these two lines of the left part of Fig. 1. On the right side of Fig. 1, line a is identical, while c represents the line from the starting point of line a along the lower base of the columella to its lateral edge with an extension to the highest point of Cupid’s bow, line b. These tension forces were derived from our own clinical experience during unilateral cleft lip repair. The measurements were taken twice per different tensile stress using an elastic stainless-steel ruler with 0.5 mm marking increments. However, measurements were only taken to an accuracy of 1 mm to reduce bias. Cadaver measurements were done exclusively by the corresponding author to reduce inter-observer variability.

**Fig. 2:**
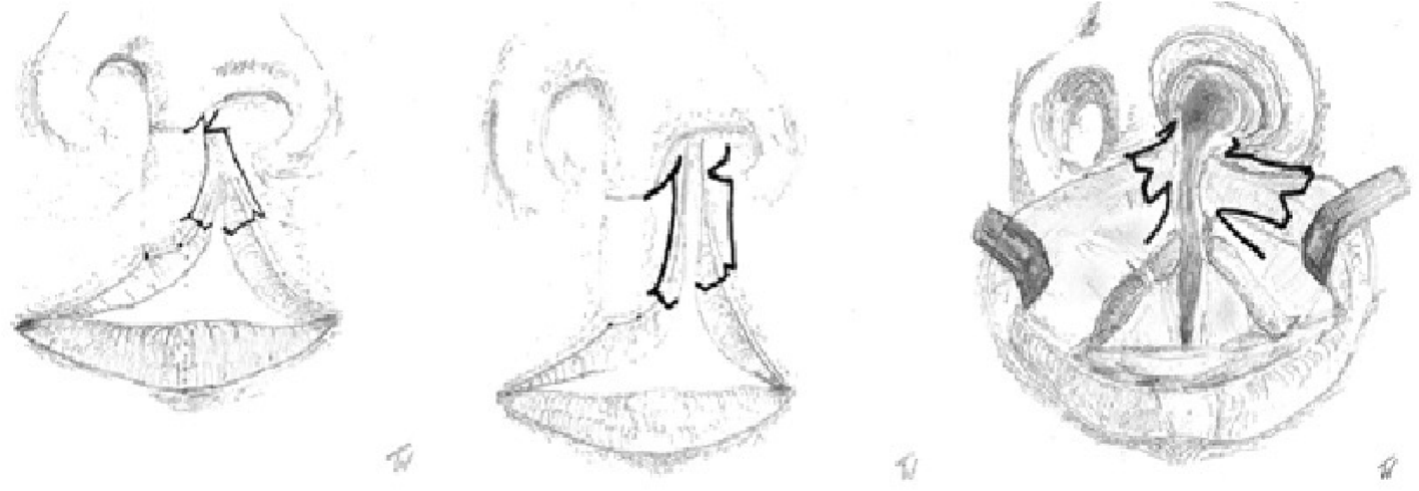
Incision pattern on a left partial cleft lip, complete cleft lip, and intraoral lines at the intraoral lip sulcus

As force increased, line a asymptotically approached line b; therefore, no further measurements of line c were conducted in the Millard group. The resulting length of the back-cut technique under tensile stress was calculated as line a, roughly derived from the sum of line b and c. Results were presented in tabular forms (see Tables 1 and 2). Data visualization and statistical analysis were performed using the software package R [13]. We found a lip lengthening effect of the back-cut incision up to 35% compared to the Millard design. Figure 2 supported by the cadaveric findings, we introduced our incision pattern in clinical practice. The Institutional Review Board classification was stated “exempt”, as known surgical techniques were used in combination.

**Table 1:** Lengthening in the craniocaudal direction of the lip with the Millard technique

Technique: Millard							
Tension force in Newton N	Line	0	1	2,5	5	Sex	Teeth
Cadaver 1 line length in mm	a	22	30	32	32	Female	no
Cadaver 1 line length in mm	b	28	32	32	32		
Cadaver 1 line length in mm	c	6					
Cadaver 2 line length in mm	a	19	23	24	24	Male	Yes
Cadaver 2 line length in mm	b	22	25	25	25		
Cadaver 2 line length in mm	c	6					
Cadaver 3 line length in mm	a	16	21	22	22	Male	Yes
Cadaver 3 line length in mm	b	18	22	22	22		
Cadaver 3 line length in mm	c	5					
Cadaver 4 line length in mm	a	18	25	26	26	Female	No
Cadaver 4 line length in mm	b	23	26	26	26		
Cadaver 4 line length in mm	c	6					
Cadaver 5 line length in mm	a	21	25	26	26	Female	No
Cadaver 5 line length in mm	b	25	27	27	27		
Cadaver 5 line length in mm	c	6					
Mean line a		19.2	24.8	26	26		
Mean line b		23.2	26.4	26.4	26.4		
SD line a		2.39	3.35	3.74	3.74		
SD line b		3.7	3.65	3.65	3.65		

Lengthening in the craniocaudal direction of the lip with the Millard technique with mean and *SD* for line a and b

**Table 2:** Lengthening in the craniocaudal direction of the upper lip with the back-cut technique

Technique: backcut-cheiloplasty							
Tension force in Newton N	Line	0	1	2,5	5	Sex	Teeth
Cadaver 6 line length in mm	a	17	30	31	31	male	no
Cadaver 6 line length in mm	b	17	21	22	22		
Cadaver 6 line length in mm	c	7					
Cadaver 7 line length in mm	a	21	33	34	34	female	no
Cadaver 7 line length in mm	b	22	24	25	25		
Cadaver 7 line length in mm	c	8					
Cadaver 8 line length in mm	a	20	33	34	34	Male	No
Cadaver 8 line length in mm	b	22	23	24	24		
Cadaver 8 line length in mm	c	7					
Cadaver 9 line length in mm	a	20	35	36	36	Female	Yes
Cadaver 9 line length in mm	b	22	24	25	25		
Cadaver 9 line length in mm	c	8					
Cadaver 10 line length in mm	a	22	36	37	37	Male	Yes
Cadaver 10 line length in mm	b	24	26	27	27		
Cadaver 10 line length in mm	c	8					
Mean line a		20	33.4	34.4	34.4		
Mean line b		21.4	23.6	24.6	24.6		
SD line a		1.87	2.3	2.3	2.3		
SD line b		2.61	1.82	1.82	1.82		

Lengthening in the craniocaudal direction of the upper lip with the back-cut technique with mean and SD for line a and b

### Patients

Patients with partial or complete unilateral cleft lip or cleft lip/alveolus/palate were selected. Surgery was performed by the first author using the technique described below. Individuals with complete cleft lip and palate were treated similarly to those with unilateral cleft lip. Postoperative follow-up adhered to our cleft team protocol. However, due to the COVID-19 pandemic, delays occurred in treatment and follow-up beginning in January 2020 (patients characteristics see below).

**Table:** 

Gender	Date of birth	Date of operation	Age at the operation (month)	Type of cleft	Follow-up on cleft team	Complications
Male	May 2023	March 2024	10	Complete lip/bone/palate left	April 2025	Complete dehiscence of the soft palate and partial dehiscence of the lip caused by an infection
Male	December 2022	August 2023	8	Partial lip/bone left	September 2023	None
Female	October 2022	August 2023	10	Partial lip/bone left	December 2024	None
Female	February 2023	November 2023	8	Complete lip/bone left	January 2024	None
Male	March 2000	February 2024	23 years	Complete lip/bone/palate right	November 2024	None
Female	January 2022	February 2023	13	Complete lip/bone/palate right	April 2024	Complete dehiscence of the soft palate and lost to follow-up since April 2024
Female	May 2022	May 2023	11	Complete lip/bone/palate left	November 2024	Postop. airway obstruction caused by a combined Furlow plasty
Male	August 2020	March 2021	11	Partial lip/bone left	February 2023	None
Female	September 2019	January 2020	3	Complete lip/bone/palate left with Simonard	December 2024	Mild hypertrophic scarring of the lip
Female	September 2019	October 2020	13	Complete lip/bone/palate left by microdeletion	January 2025	None
Female	December 2021	August 2022	7	Partial lip/bone left	June 2024	None
Female	February 2020	February 2021	11	Complete lip/bone with ocular hypertelorism	August 2023	Hypertrophic scarring as a consequence of a postoperative wound infection of the lip with sec. corrections, lost to follow-up since October 2023
Male	May 2020	November 2024	5	Partial lip left	November 2022	None
Male	November 2019	November 2024	12	Partial lip/bone right	February 2025	None
Male	November 2003	January 2024	20 years	Complete lip/bone right	January 2024	Lost to follow-up since January 2024
Male	March 2024	January 2025	10	Bilateral complete lip/bone/palate	February 2025	None
Male	July 2024	Februayr 2025	6	Complete lip right	March 2025	None

### Drawings and surgical technique

**Fig. 3:**
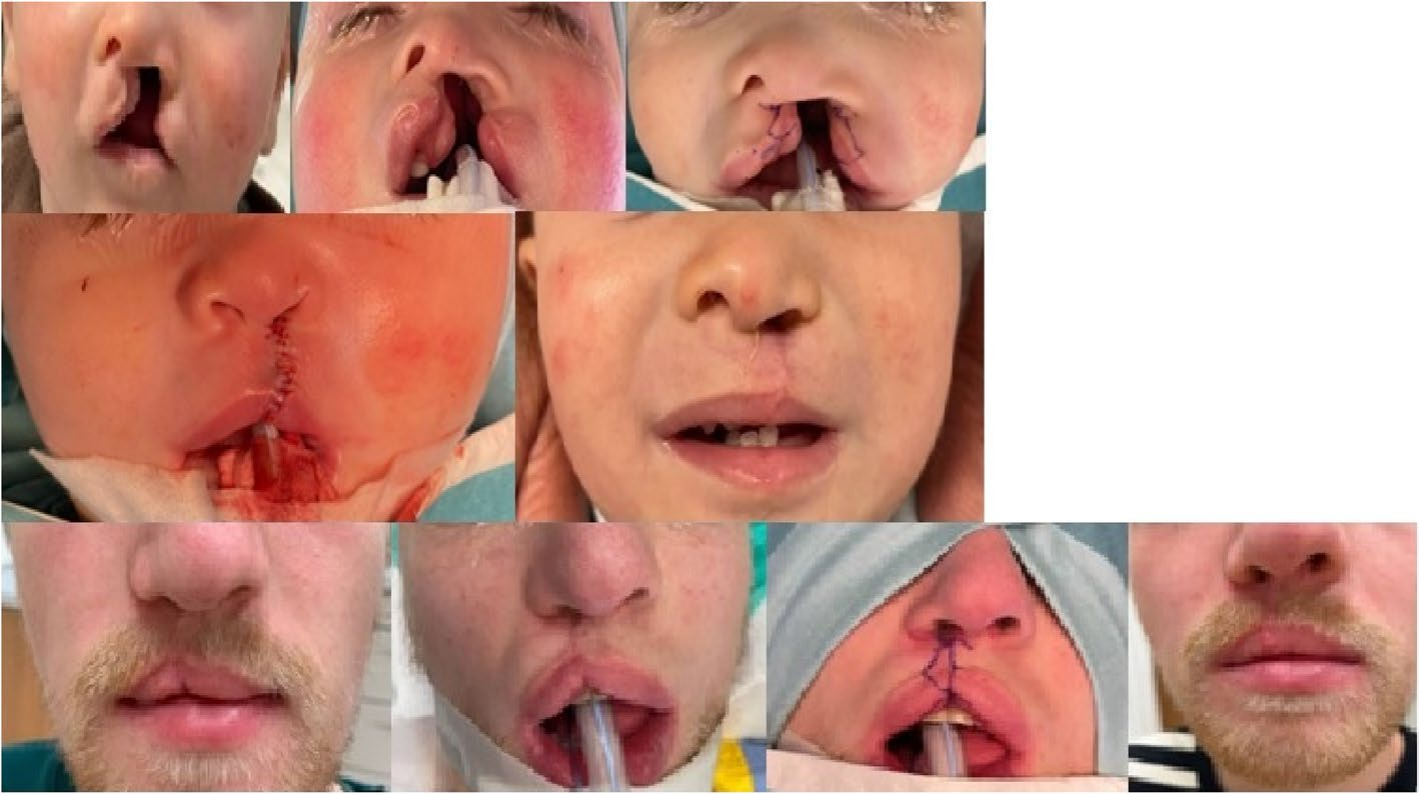
Above: complete cleft lip and palate left at 4 months of age with presurgical taping, intraoperative drawings with immediate result and 6 months postoperative at the age of 1.5 years with a postoperative wound infection caused by continuously nostril retainer manipulation by the child and partial dehiscence at the alar base with scar contraction. Below: Z-back cut cheiloplasty at the age of 24 with preoperative, intraoperative, and postoperative photos at 6 months including the drawing pattern

**Fig. 4:**
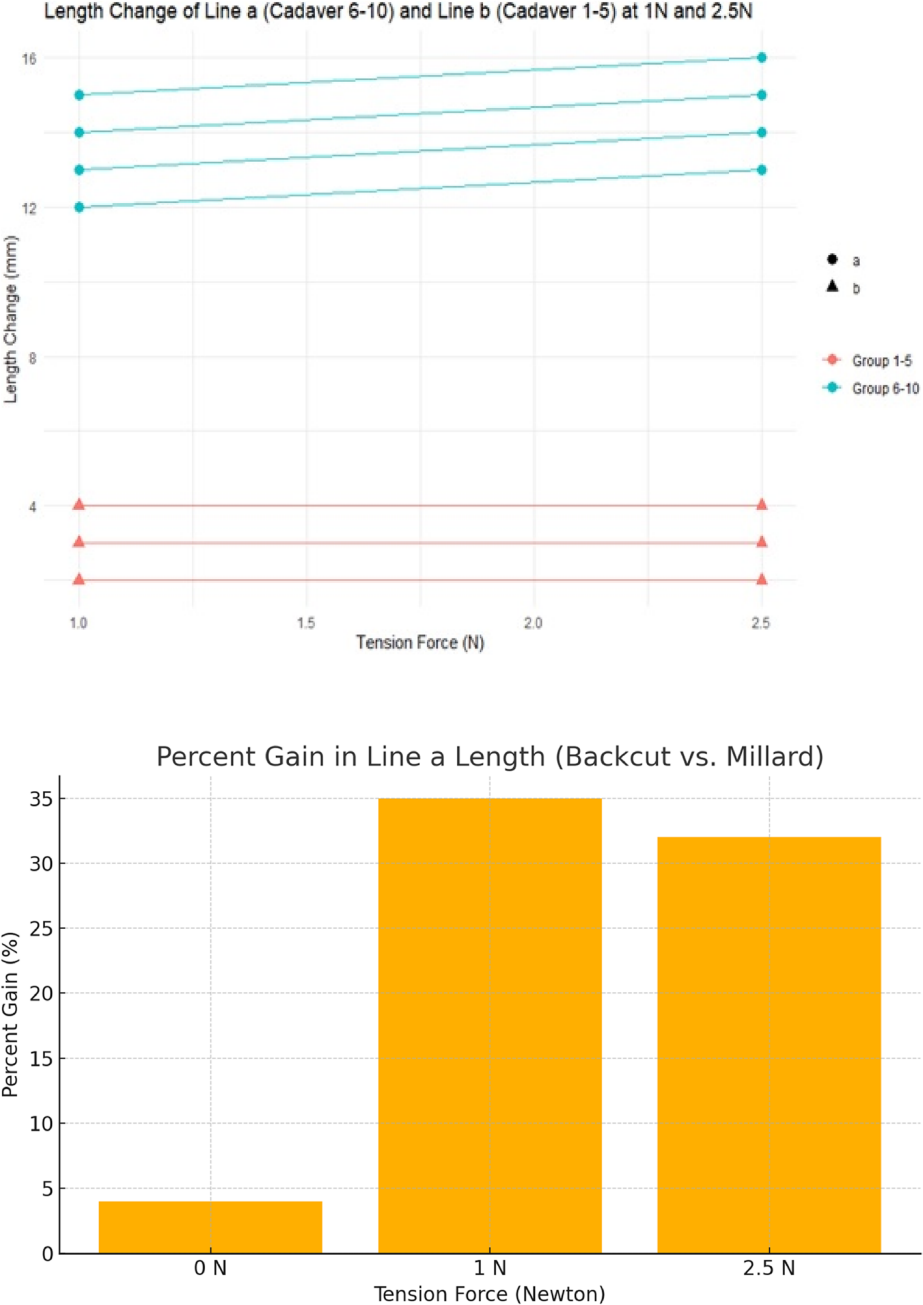
No increase in the length chance of line a in Millard’s incision line. Increase of lengthening of line a in the group of back-cut incision compared to line b in Millard design with 1N and 2.5N tension force. No further lengthening of both lines with 5N. With increasing tension, line a approximates line b in Millard’s technique, while lines b+c approximates line a in the back-cut technique. Lower Part: Percentage gain in length between the two incision patterns

**Fig. 5:**
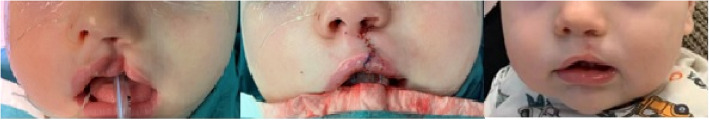
Operated upper lip at the age of 9 months, intraoperative result and 7 weeks postoperatively with our technique by partial cleft of the lip and alveolus

**Fig. 6:**
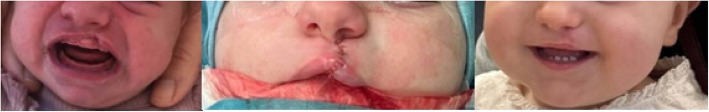
Partial cleft of the left upper lip with closure by Z-back cut-cheiloplasty preoperatively at 2 weeks of age, direct intraoperatively at the age of 10 months and at 6 weeks postoperatively

The incision design is illustrated in Fig. 2. Initial steps include marking of Cupid’s bow, the subnasal, the lateral base of the columella on the cleft side, and an additional perpendicular marking 2 mm cranial to the cleft-side peak of Cupid’s bow in the white roll of the vermilion border under loupe magnification. From this point, a line is drawn just outside the vermilion border toward the nasal sill, with a back cut extending to the marked lateral columella base. From the Noordhoff point (the point along the lateral lip where the vermillion height is at its greatest and the white roll is well formed but becomes less distinct toward the cleft as the progressive deficiency of the orbicularis oris muscle), the incision is extended outside the vermilion border towards the cleft sill, incorporating a small Fisher’s triangle (as small triangular pennant skin flap above of Noordhoff point) cranially in the lateral white roll. A medial triangular skin flap is added at the nostril base. The dry/wet line of the vermilion is marked bilaterally. Small distal triangular flaps of the dry vermilion skin are created for additional lip volume. For complete clefts, two mucosal vermilion flaps are dissected to close the nasal floor. In incomplete clefts, they can be discarded. The orbicularis oris muscle and its subdivisions, such as the depressor septum nasalis muscle, are released from the base of the columella, nasal ala, and the alveolar cleft of the maxillary bony segment with a single or double mucosal incision at the oral vestibular sulcus. The vestibular mucosa is sutured with interrupted Vicryl 4.0 sutures, followed by cranial-to-caudal adaptation of the orbicularis oris muscle with PDS 4.0 and Vicryl 4.0 sutures. Skin closure begins at the vermilion border using Monocryl 6.0. The contralateral relaxing back-cut incision is positioned precisely in the sulcus of the contralateral white roll for Fisher’s pennant flap placement. The small flap is trimmed and sutured into place, followed by nostril closure. The triangular flaps of the Z-plasty at the columella base are adjusted to size before final closure. Excess mucosa is resected before placing steristrips across the suture line. In complete cleft lip and palate cases, primary rhinoplasty following McComb’s method (with blunt mobilization of the nasal skin around the lower latera) is performed, with a silicone nostril retainer placed for 6 to 8 weeks. The technique may also be suitable for secondary lip revision in adults (see Figs. 3, 4, 5, and 6). To date, a total of 17 patients with a partial or complete cleft lip have been operated on using the technique described here in the last 4 years. So far, one secondary revision has been performed; according to our cleft team protocol, these are in most cases-only performed in adolescence, sometimes in combination with rhinoplasty.

Our tension force studies demonstrated insufficient lengthening of Millard’s advancement-rotation flap compared to our back-cut incision (see Fig. 4). Between 2.5 N and 5 N tension, no further lengthening occurred; thus, statistical analysis at 5 N was omitted. Comparisons between the two incision patterns at 1 N and 2.5 N showed no significant elongation with Millard’s incision, whereas the back-cut technique produced a significant increase. The results were highly statistically significant (*p* < 0.001, Student’s *t*-test). This suggests that Millard’s technique does not appear to achieve the expected skin lengthening via rotation and advancement. In contrast, an incision along the philtral column with a back-cut at the cleft-side columella base, as part of a Z-plasty, significantly enhances lengthening. Furthermore, the resulting straight line reassembles the lateral border of the philtrum more accurately. The resulting defect of the back-cut incision at the lateral base of the columella can be filled up with the cutaneous triangle of the contralateral side of the cleft as part of a Z-plasty.

## Discussion

Our study correlates incision-line lengthening in unilateral cleft lip repair with applied tension forces. The primary and most important limitation is the use of adult cadaveric skin. However, we believe the lengthening effect is largely independent of age, as it is based on the geometric principle of Z-plasty [14, 15]. The data indicate that the distance between the columella base and Cupid’s bow lateral border depends on the incision pattern. This tension line forms the future philtral column on the cleft side post-surgery. To maximize lengthening, we recommend combining Z-plasty with Fisher’s pennant flap and relaxing incisions at the gingival sulcus to prevent upper lip under-projection. We also use vermilion mucosal flaps in complete cleft lip and palates to reconstruct the nasal floor and to augment shortness of mucosal lining at the site of the cleft sulcus. As known in Furlow’s palate closure technique or constriction ring syndromes, the Z-plasty principle adds lengthening and less chance for retraction in the long run. The meticulous adapting of all parts—but especially the caudal part—of the upper orbicularis oris muscle seems the most eminent step in cleft lip closure to prevent vermilion notching or even whistling deformity [16] in combination with adequate lengthening of the inner mucosal lining of the cleft repair. The resulting straight line reassembles the lateral border of the philtrum more accurately. The resulting defect of the back-cut incision at the lateral base of the columella can be filled up with the cutaneous triangle of the contralateral side of the cleft as part of a Z-plasty. Furthermore, the central base of the columella stays untouched and free of visible scars. Over the past 4 years, we have performed our technique on 17 patients. Although long-term results remain pending, we hope to expect less secondary lip revisions due to reduced vertical scar contracture. We are well aware that cadaveric skin behaves differently from pediatric skin and therefore it is difficult to draw direct conclusions from our cadaver study. Follow-up of these children is a prerequisite to estimate the value of our developed technique. We also believe our technique is well-suited for bilateral cleft lip repair due to its additional philtrum lengthening capabilities. This novel incision pattern has not been previously published and represents a valuable addition to existing techniques by incorporating the best elements from previous approaches.

## Data Availability

No datasets were generated or analysed during the current study.
